# Insight into the mechanism of gallstone disease by proteomic and metaproteomic characterization of human bile

**DOI:** 10.3389/fmicb.2023.1276951

**Published:** 2023-12-04

**Authors:** Xue-Ting Yang, Jie Wang, Ying-Hua Jiang, Lei Zhang, Ling Du, Jun Li, Feng Liu

**Affiliations:** ^1^Minhang Hospital, Fudan University, and Shanghai Key Laboratory of Medical Epigenetics, The International Co-laboratory of Medical Epigenetics and Metabolism, Ministry of Science and Technology, Institutes of Biomedical of Sciences, Fudan University, Shanghai, China; ^2^Key Laboratory of Digestive Cancer Full Cycle Monitoring and Precise Intervention of Shanghai Municipal Health Commission, Minhang Hospital, Fudan University, Shanghai, China; ^3^Department of Surgery, Tongren Hospital, Shanghai Jiaotong University School of Medicine, Shanghai, China

**Keywords:** bile, metaproteomics, gallstone, microbiome, proteomics, cholecystitis

## Abstract

**Introduction:**

Cholesterol gallstone disease is a prevalent condition that has a significant economic impact. However, the role of the bile microbiome in its development and the host’s responses to it remain poorly understood.

**Methods:**

In this study, we conducted a comprehensive analysis of microbial and human bile proteins in 40 individuals with either gallstone disease or gallbladder polyps. We employed a combined proteomic and metaproteomic approach, as well as meta-taxonomic analysis, functional pathway enrichment, and Western blot analyses.

**Results:**

Our metaproteomic analysis, utilizing the lowest common ancestor algorithm, identified 158 microbial taxa in the bile samples. We discovered microbial taxa that may contribute to gallstone formation, including β-glucuronidase-producing bacteria such as Streptococcus, Staphylococcus, and Clostridium, as well as those involved in biofilm formation like Helicobacter, Cyanobacteria, Pseudomonas, *Escherichia coli*, and Clostridium. Furthermore, we identified 2,749 human proteins and 87 microbial proteins with a protein false discovery rate (FDR) of 1% and at least 2 distinct peptides. Among these proteins, we found microbial proteins crucial to biofilm formation, such as QDR3, ompA, ndk, pstS, nanA, pfIB, and dnaK. Notably, QDR3 showed a gradual upregulation from chronic to acute cholesterol gallstone disease when compared to polyp samples. Additionally, we discovered other microbial proteins that enhance bacterial virulence and gallstone formation by counteracting host oxidative stress, including sodB, katG, rbr, htrA, and ahpC. We also identified microbial proteins like lepA, rtxA, pckA, tuf, and tpiA that are linked to bacterial virulence and potential gallstone formation, with lepA being upregulated in gallstone bile compared to polyp bile. Furthermore, our analysis of the host proteome in gallstone bile revealed enhanced inflammatory molecular profiles, including innate immune molecules against microbial infections. Gallstone bile exhibited overrepresented pathways related to blood coagulation, folate metabolism, and the IL-17 pathway. However, we observed suppressed metabolic activities, particularly catabolic metabolism and transport activities, in gallstone bile compared to polyp bile. Notably, acute cholelithiasis bile demonstrated significantly impaired metabolic activities compared to chronic cholelithiasis bile.

**Conclusion:**

Our study provides a comprehensive metaproteomic analysis of bile samples related to gallstone disease, offering new insights into the microbiome-host interaction and gallstone formation mechanism.

## 1 Introduction

Cholesterol gallstone disease is a prevalent condition and a significant economic burden worldwide. The prevalence rates of gallstone disease vary across regions, with approximately 30–40% in South America, around 20% in Europe, and 5–8% in Asia (Chen et al., 2006; Lammert et al., 2016; Li and Gao, 2019). Gallstones can occur in various parts of the human biliary system, including the gallbladder, extrahepatic bile duct, or intrahepatic duct. Gallstones are commonly classified as either cholesterol or bilirubin gallstones, with cholesterol gallstones being the most predominant type. While the exact cause of gallstone formation remains unclear, various factors contribute to the development of gallstone disease, including genetic predisposition, cholesterol crystallization, and bile supersaturation. Additionaly, several risk factors have also been identified, such as fatty liver, diabetes, obesity, and dysbiosis of the microbiome (Di Ciaula et al., 2018). Notably, microbial infections and the interaction between the host and the microbiome play a significant role in the development of cholecystitis (CHL) and gallstone disease (Grigor'eva and Romanova, 2020).

Dysbiosis, characterized by altered bacterial composition and abundance, has been implicated in the pathogenesis of various diseases, including inflammatory conditions, cancers, cardiovascular diseases, and metabolic disorders like diabetes and obesity (Thursby and Juge, 2017). To investigate the changes in the microbiota and its association with gallstone formation, researchers have utilized 16S rRNA gene sequencing to analyze microbial components in bile, gut, and gallstones of patients with cholelithiasis (Shen et al., 2015; Kose et al., 2018; Molinero et al., 2019). The microbiota in the gut, biliary tract, and gallbladder may contribute to gallstone development. For instance, studies have demonstrated that gut microbes, such as Desulfovibrionales, can influence the formation of cholesterol-type gallstones by regulating bile acid production and cholesterol secretion (Hu et al., 2022). Furthermore, dysbiosis in the biliary microbiota has been associated with the recurrence of bile duct stones (Tan et al., 2022). Specific components of the bile microbiome, such as Enterobacteriaceae, have also been linked to the development of CHL and gallbladder cancer (Choe et al., 2021). Therefore, 16S rRNA gene sequencing serves as a valuable tool for identifying and assessing the relative abundance of microbiota in bile samples, including at the species level, in both diseased individuals and healthy controls. It is important to acknowledge that DNA sequencing technologies alone cannot directly elucidate the protein function of microbial communities within these bile samples.

Mass spectrometry (MS)-based proteomics has emerged as a powerful tool for investigating protein expression patterns in human samples. Several proteogenomic studies have been performed to analyze the composition of human protein in bile samples obtained various clinical conditions, such as sclerosing cholangitis (Reinhard et al., 2012; Rupp et al., 2018), biliary stenosis (Farina et al., 2009), cholangiocarcinoma (Kristiansen et al., 2004; Farid et al., 2011; Lankisch et al., 2011; Shen et al., 2012; Navaneethan et al., 2015; Laohaviroj et al., 2017; Voigtlander et al., 2017; Ren et al., 2019; Son et al., 2020), gangrenous CHL (without gallstones) (Wang L. et al., 2018) and gallstone disease (Zhou et al., 2005; Barbhuiya et al., 2011; Zhang et al., 2013). However, these studies were constrained by the utilization of pooled-sample strategies, low throughput methods, or outdated platforms and technologies, which limited the scope of protein identification. Recently, a study analyzed the bile metaproteome of gallbladder cancer, gallstones, and healthy controls (Sharma et al., 2022). Although the study successfully explored microbial taxa based on microbial peptides, further investigation into microbial proteins remains incomplete.

In the present population-scale study, we conducted a comprehensive proteomic and metaproteomic analysis of gallbladder bile samples obtained from 40 patients diagnosed with gallstone disease and gallbladder polyp. We employed trapped ion mobility spectrometry coupled with time-of-flight mass spectrometry (TIMS-TOF-MS) to explore the microbial composition and microbiome characteristics linked to gallstone disease, as well as the host immune responses that could contribute to gallstone formation and cholelithiasis.

## 2 Materials and methods

### 2.1 Human bile sample collection

A total of 40 human bile samples were analyzed, consisting of 31 gallstone bile samples and 9 control bile samples (gallbladder polyp or normal bile). Among the 31 gallstone patients, 14 had acute CHL, while 17 had chronic CHL (Supplementary Table S1). The 9 gallstone-free controls consisted of individuals with polyps but no visible gallstone or CHL symptoms. Detailed information regarding the samples can be found in Supplementary Table S1. The bile samples from patients diagnosed with cholesterol gallstone disease were collected at the Department of General Surgery of Shanghai Tongren Hospital (Shanghai, China). Control bile samples were obtained from individuals with gallbladder polyps who underwent thorough examinations to confirm the absence of gallstones and gallbladder inflammation. Bile extraction from the gallbladder was performed during cholecystectomy using a sterile syringe, and subsequently, the samples were stored at −80°C until further analysis. Written consent was obtained from all patients or their relatives, and the study was approved by the Institutional Research Ethics Committee of the Institutes of Biomedical Sciences, Fudan University.

### 2.2 Experimental design and statistical rationale

The proteins from each sample were separated using SDS-PAGE, followed by in-gel tryptic digestion. The resulting digestion products were then subjected to analysis using reverse-phase liquid chromatography-mass spectrometry (RPLC–MS). A total of 40 LC–MS analyses were conducted.

### 2.3 Extraction of bile proteins

To extract proteins from the bile samples, 200 μL of raw bile was mixed with 400 μL of chloroform and 10 μL of acetic acid. The mixture was vortexed for 1 min and then chilled on ice. This cycle was repeated 10 times. Subsequently, the tube was centrifuged at 12,000 rpm for 10 min at 4°C, and the enriched lower organic phase containing bilirubin was discarded. The upper and middle layers containing proteins were collected and mixed with chloroform and acetic acid. The extraction and centrifugation steps were repeated as described above. The resulting supernatant was then added to 1.2 mL of ice-cold acetonitrile (ACN) and placed in a −20°C freezer to promote precipitation. After centrifugation at 12,000 rpm for 10 min at 4°C, the obtained pellet was suspended with 1 mL of ice-chilled acetone and sonicated to ensure complete suspension. Subsequently, the tube was centrifuged, and the pellet was suspended and washed with 1 mL of isopropanol. Another round of centrifugation was performed, followed by resuspension and washing with 1 mL of dehydrated alcohol. Finally, the resulting pellet was dissolved using 100 μL 0.5 M triethylammonium bicarbonate buffer (TEAB, cat no. T7408, Sigma Life Science, Shanghai, China) with 0.1% SDS, aided by sonication. Protein concentrations were determined using the BCA protein assay kit (Beyotime, LTD Inc., Shanghai, China) according to the manufactory’s instructions.

### 2.4 SDS-PAGE and in-gel tryptic digestion

**Figure 1:**
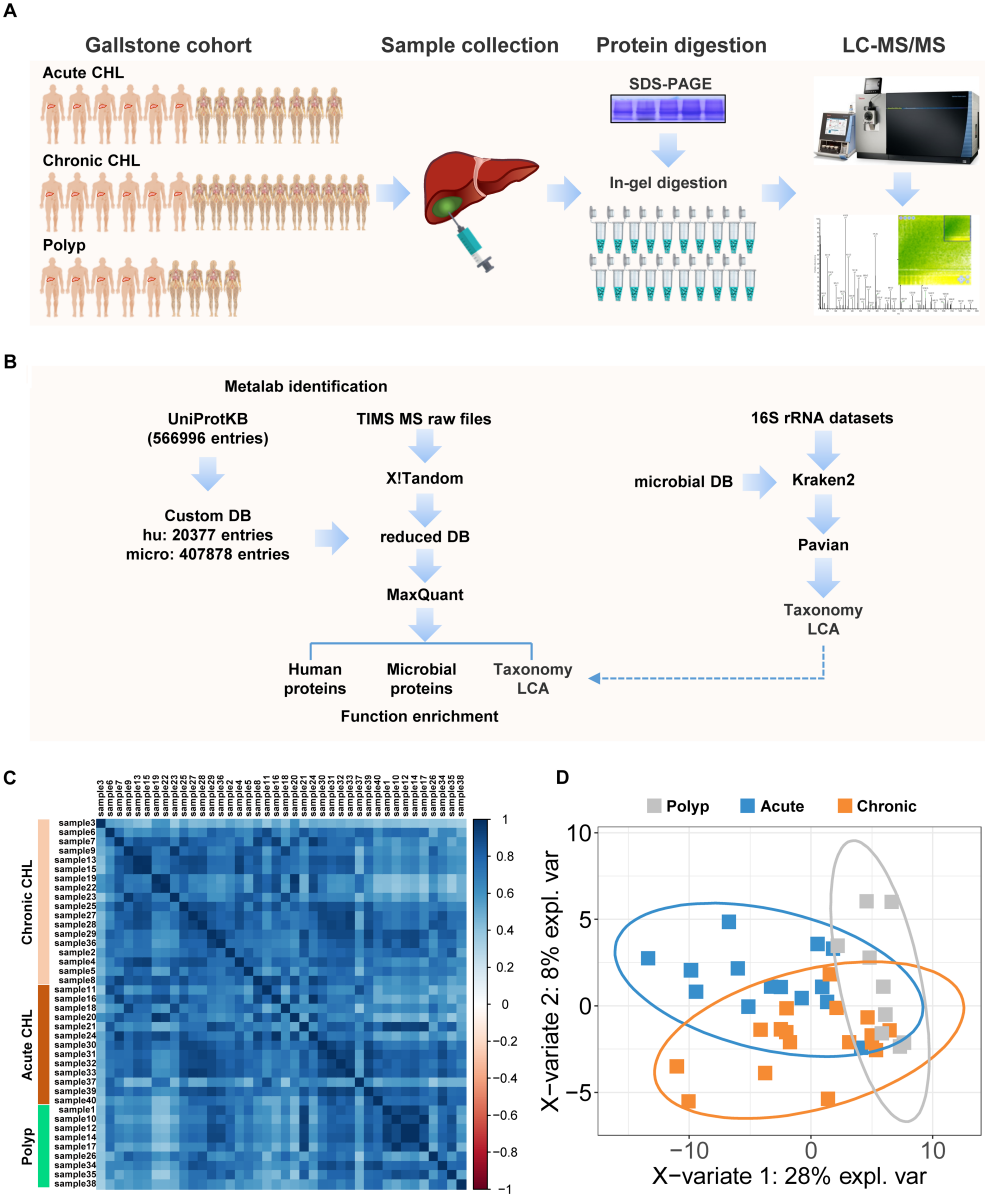
Proteomic and metaproteomic analysis of human bile with or without gallstone. **(A)** The cohort enrolled in this study contains 14 acute CHLs, 17 chronic CHLs and 9 gallstone-free patients with gallbladder polyps. The bile fluids were collected during gallbladder resection of gallstone patients or polyp remove of patients. The proteins were extracted and separated using SDS-PAGE, followed by in-gel tryptic digestion. LC–MS analysis was performed using TIMS-TOF pro mass spectrometer. **(B)** The peptides were identified by two-step database searching using Metalab against a comprehensive protein database (UniProtDB) and the peptides were further subjected to taxonomic lowest common ancestor (LCA) analysis. Metataxonomic analysis using public 16S rRNA datasets of human bile by Kraken2 program was performed independently to verify the metaproteomic results. **(C)** The correlation plot of the bile samples based on protein identification. **(D)** PLS-DA of bile samples based on identified microbial taxa. The ellipses illustrated the confidence interval.

The proteins of the mixed samples (30 μg) were separated using mini-size 10% SDS-PAGE. Each lane was cut into one slice (Figure 1) and subjected to in-gel tryptic digestion, following a previously described protocol with slight modifications (Cui et al., 2013). Gel slices were cut into approximately 1 mm^3^ granules using a surgical knife. A destaining solution containing 50% ACN was added to the tube, which was then agitated at 1,250 rpm for 5–10 min. Subsequently, the tube was centrifuged and the supernatant was discarded. This destaining step was repeated three times to ensure the removal of residual dye. The gel granules were shrunk by adding 1 mL of 100% ACN and agitated. The tube was then centrifuged, and the supernatant was aspirated. The granules were vacuum dried for 20 min. For the reduction reaction, 300 μL of freshly prepared 64 mM DTT/25 mM NH_4_HCO_3_ was added, followed by incubation at 56°C for 1 h with gentle agitation. The supernatant was aspirated, and the free sulfhydryl groups were blocked with 300 μL iodoacetamide (IAA) solutioin (130 mM in 25 mM NH_4_HCO_3_) for 45 min at room temperature in a dark place. The tubes were centrifuged to remove the supernatant, and the pellets were incubated in 500 μL of 25 mM NH_4_HCO_3_ for 3 min with agitation. The supernatant was removed by centrifugation, and the pellets were shrunk using 100% ACN. The granules were then swollen with the enzyme mixture containing 16 ng/μL trypsin (cat no. V5111, Promega, Beijing, China) and 3 ng/μL rLysC (HLS LYS001C, HUA LISHI SCIENTIFIC, Beijing, China). Excess enzyme solution was removed, and digestion was carried out overnight at 37°C with gentle agitation. After digestion, 300 μL of 50% ACN/5% formic acid (FA) solution was added to the tubes, which were incubated in a water bath with sonication for 10–20 min. Following centrifugation, the supernatant was collected. The extraction step was repeated four times, and the supernatants were combined and lyophilized.

### 2.5 Desalting

The peptides were dissolved in 100 μL of 0.1% FA and desalted using MonoSpin C18 (Cat. No. 5010–21701, GL Sciences Inc.) according to the manufacturer’s instruction with minor modifications. In brief, the blank spin column was conditioned with 100 μL of 100% ACN/0.1% FA solution, followed by a conditioning step with 200 μL of water. Next, the 100 μL peptide sample was loaded onto the spin column for adsorption. After centrifugation to remove the flow-through, the spin column was washed with 100 μL of water. Subsequently, the clean peptides were eluted with 150 μL of 50% ACN/0.1% FA solution and lyophilized.

### 2.6 Peptide quantitation

Peptide quantification was conducted employing the Pierce Quantitative Colorimetric Peptide Assay kit (Pierce# 23275, Thermo Fisher Scientific, Shanghai, China), following the manufacturer’s guidelines. We used standard peptides to create a dilution series for generating a standard curve. Peptide samples underwent a 10-fold dilution with ultrapure water. The reaction process involved the addition of working reagents, followed by an incubation period of 15 min at 37°C. We measured the absorbance at a wavelength of 480 nm using a microplate reader. The concentration of peptides in each sample was calculated referencing the standard curve. Lastly, we adjusted the concentration of each peptide sample was adjusted to 200 ng/μL before proceeding with mass spectrometry analysis.

### 2.7 Label-free LC–MS analysis of bile proteome

Bile protein digests from each sample were subjected to a single-shot analysis using a TIMS-TOF Pro mass spectrometer (Bruker Daltonics GmbH, Bremen, Germany) equipped with a Nano electrospray ionization (ESI) source. We separated 200 ng of peptides using an Ionopticks Aurora Series emitter column (25 mm x 75 μm ID, 1.6 μm C18). The mobile phase A (H_2_O/0.1% formic acid) and mobile phase B (100% acetonitrile/0.1% formic acid) were delivered through a liquid chromatograph nanoElute (Bruker Daltonics GmbH, Bremen, Germany) at a flow rate of 200 nL/min, maintaining the column temperature at 50°C. The 30-min gradient elution program was set as follows: an increment from 5% B to 22% B over 20 min, a rise to 37% B for 4 min, a subsequent increase to 80% B for 2 min, followed by an isocratic hold at 80% B for 4 min. The survey MS scans ranging from m/z 100 to 1,700 were succeeded by 10 Parallel Accumulation-Serial Fragmentation (PASEF) tandem MS scans with a cycle time of 1.16 s. The intensity threshold for online PASEF analysis was set at 5000, with the accumulation and ramp times fixed at 100 ms. The ion source voltage and temperature were 1,500 v and 180°C, respectively. The drying gas flow rate was set to 3 L/min, and the ionic mobility range was configured to 0.7–1.3 *Vs*/cm^2^.

### 2.8 Metaproteomic database searching

The raw data generated by TIMS-TOF Pro were converted into mxXML files using ProteoWizard Version: 3.0.21352-04cc5bb^1^ (Chambers et al., 2012). These mxXML files served as input for the downstream metaproteomic database searching, which was automated using Metalab version 2.2.1 (Cheng et al., 2017). Metalab incorporates X!Tandem (version 20151215) and MaxQuant (version 1.6.5.0) for the first and second round of database searching, respectively (Craig and Beavis, 2004; Tyanova et al., 2016). In the first round of X!Tandem searching, the database search was conducted against a custom microbial database (407,878 entries) comprising protein sequences from Bacteria, Virus, Archaea, and fungus. These sequences were extracted from the UniProtKB/Swiss-Prot database of Release 2022_01 (566,996 entries). To construct the custom database, we obtained all taxonomy IDs associated with Bacteria (taxon id 2), Virus (taxon id 10239), Archaea (taxon id 2157) and Fungus (taxon id 4751) using NCBI Entrez Edirect. The protein sequences were then extracted from the UniProtKB/Swiss-Prot database based on the organism annotation (“OX”) provided in each sequence title. The database search was performed in parallel against the Human (taxon id 9606) protein database, which comprised 20,377 sequences extracted from UniProtKB/Swiss-Prot database of Release 2022_01. The fixed post-translation modification (PTM) was carbamidomethylation of cysteine (C), while the variable modifications included acetylation of protein N-terminus, deamidation of asparagine (N) and glutamine (Q), and oxidation of methionine (M). The enzyme used was trypsin, allowing for two missed cleavage sites. No spectra clustering was performed. A sample-specific database was generated at the end of X!Tandem searching.

In the second round searching, MaxQuant was utilized to search against the reduced sample-specific database. The false discovery rates (FDR) for peptide-spectrum matches (PSMs), protein, and modification site was set at 0.01. The search was operated in parallel using revert decoy mode and included contaminant sequences. The match-between-run function was applied with the following parameters: Match ion mobility window, 0.05; Alignment time window, 20 min; Alignment ion mobility, 1; Match unidentified features, true. Protein expression was quantified using the label-free quantification (LFQ) module, with the following parameters: LFQ min. Ratio count, 1; Label min. Ration count, 1. Proteins that shared the same set of peptides were automatically grouped into a protein group. Default settings were accepted for all other parameters not specified here. The resulted proteinGroups.txt file was filtered using R scripts, and proteins labeled as “Potential contaminant,” “Reverse” or “Only identified by site” were discarded. Additionaly, proteins with fewer than 2 unique peptides were discarded.

Using the peptides identified by MaxQuant, Metalab performed taxonomic lowest common ancestor (LCA) analysis against the built-in taxonomic database. The analysis revealed the top nodes, including Eukaryota, Bacteria, Archaea, and Viruses. Taxa were determined based on a minimum of three unique peptide counts. The quantification of each taxonomic node was performed using the LFQ value.

### 2.9 Functional enrichment analysis

We obtained the Gene ontology (GO) annotations for all proteins from the Uniprot database.^2^ To performed function enrichment analysis of differential human proteins, we utilized g:Profiler^3^ (Raudvere et al., 2019). In the g:Profiler analysis, the Benjamini-Hochberg FDR method was employed for multiple testing correction, with a threshold set at 0.0001. No electronic GO annotations were allowed. We analyzed the Gene ontology of molecular function (GO_MF), cellular component (GO_CC), and biological process (GO_BP), as well as the biological pathways of KEGG, Reactome (REAC), and WikiPathways (WP).

### 2.10 Linear discriminant analysis effect size analysis

To identify the key phyla that contribute significantly to the differences between gallstone disease and polyps, we conducted linear discriminant analysis effect size (LEfSe) analysis using the online Galaxy pipeline.^4^ The factorial Kruskal-Wallis test was employed to calculate LDA Effect Size, with an alpha value of 0.05 for the test among classes. Discriminative features were determined based on a logarithmic LDA score threshold of 2.0. Prior to the analysis, the LFQ-intensity values were converted to percentages. This conversion involved subtracting the LFQ-intensity value of a taxon from the total sum of LFQ-intensity values of all taxa in a specific sample. In cases where values were missing, they were substituted with zero.

### 2.11 Other bioinformatic analyses

Pearson correlation between bile samples were analyzed using the R package Corrplot v0.84. For hierarchical clustering and heatmap generation, the R package pheatmap v1.0.12 was employed with Euclidean distance and complete linkage. To conduct partial Least-Squares Discriminant Analysis (PLS-DA) using taxonomic data, we utilized R package mixOmics v6.22.0.

### 2.12 Metagenomic analysis of 16S rRNA sequencing datasets

We downloaded the raw sequences (filtered and clipped, fastq format) of six datasets of 16S rRNA sequencing from the Sequence Read Archive (SRA) of NCBI database. The accessions for these datasets were PRJNA439241, PRJEB15501, PRJEB12755, PRJNA580086, PRJNA793871, and PRJNA543184. These datasets consisted of 27, 126, 59, 45, 122, and 100 human bile samples, respectively, which were subjected to 16S rRNA gene sequencing (Supplementary Table S3). To performed taxonomic classification, we utilized Kraken2 program^5^ and matched the sequences against the Kraken2-microbial database (September 2018, 30GB) (downloaded from https://lomanlab.github.io/mockcommunity/mc_databases.html). This database contained genomic sequences from archaea, bacteria, fungi, protozoa, viral and Univec_Core. Sequences from taxa other than bacteria were served as a performance control, ensuring that the 16S rRNA sequencing reads would not identify viral hits. The Kraken2-microbial database consisted of a total of 21,207 taxonomy nodes and had a table size of 5,631,915,627. Kraken2 employs an exact k-mer method to accurately and efficiently match and classify reads to the lowest common ancestor (LCA) of microbial genomes (Lu et al., 2022). The Kraken2 reports from different samples were further analyzed and integrated using Pavian^6^ (Breitwieser and Salzberg, 2020).

### 2.13 Dot blotting analysis

Proteins from 5 μL of bile samples were transferred to PVDF membrane (Merck Millipore, Shanghai, China) using the Bio-Dot microfiltration apparatus according to the manufacturer’s instructions (Bio-Rad Laboratories, Inc., United States). The immunoblot was conducted according to previously described methods (Wang et al., 2021). The CFI rabbit polyclonal antibody (Cat. XC5623) and C5 rabbit polyclonal antibody (Cat. XC8104) were purchased from Shanghai Outdo Biotech Co., Ltd.

### 2.14 Availability of data and materials

The raw MS data, which underpins the findings of this article, can be accessed at the ProteomeXchange Consortium^7^ via the iProX partner repository.^8^ The dataset identifier for this study is IPX0004827000 or PXD035915. Additionally, the dataset that supports the conclusions of this article is included within the article itself.

### 2.15 Statistics

Statistical significance was determined using a two-sided Welch’s t test. A *p*-value < 0.05 was considered statistically significant. For the g:Profiler analysis, the Benjamini-Hochberg FDR method was employed for multiple testing correction, with a threshold set at 0.0001. All measured values were reported as mean ± standard deviation (SD).

## 3 Results

### 3.1 Proteomics and metaproteomic analyses of human bile from gallstone and gallstone-free patients

We performed a comprehensive analysis of 40 human bile samples obtained from gallstone patients and gallstone-free controls (Figure 1A). To facilitate the analysis, we employed a two-stage database search strategy using the MaxQuant workflow integrated into the Metalab program (Cheng et al., 2017), as depicted in Figure 1B. In the first round of searching, we searched over 2.6 million MSMS spectra against the primary database. This first-stage searching identified 30,575 protein sequences, which were subsequently used as a secondary database for the second round searching by MaxQuant (Tyanova et al., 2016). MaxQuant identified 23,033 peptides, which were then subjected to taxonomy LCA analysis by Metalab. Ultimately, we identified 2,836 protein groups, including 87 microbial and 2,749 human proteins. The correlation coefficients for the bile components between polyp samples were found to be 0.75 ± 0.16. Similarly, the correlation coefficients between chronic CHL samples were 0.73 ± 0.13, while the correlation coefficients between acute CHL samples were 0.72 ± 0.15. These results indicate that there is a greater degree of variation in individual bile components within samples from patients with CHL compared to the samples from individuals with polyps (Figure 1C).

### 3.2 Microbial taxa in human gallstone bile identified by microbial peptides

**Figure 2:**
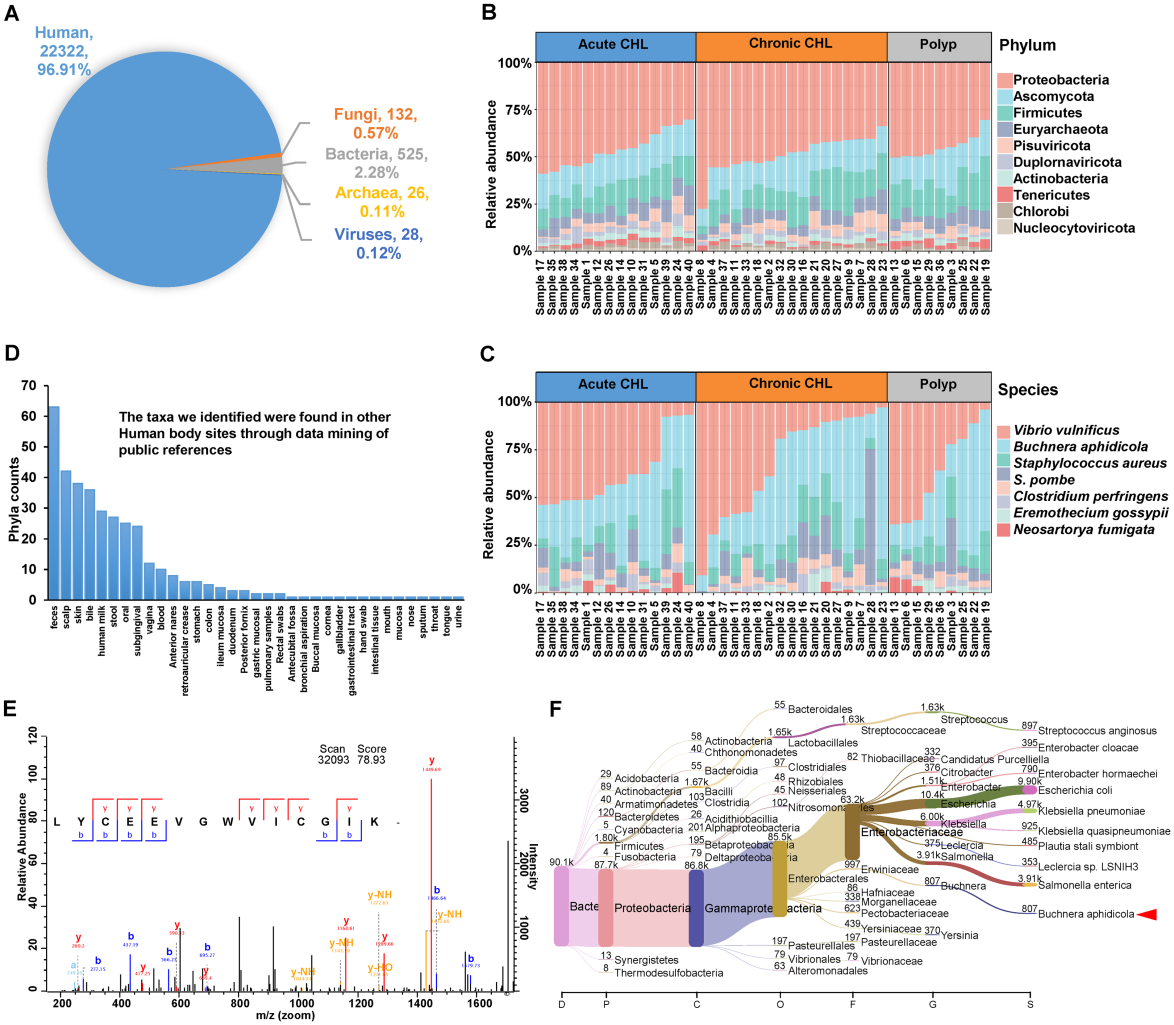
Identification of microbes from gallstone and polyp bile samples. **(A)** The proportion of unique peptides identified in human, fungi, bacteria, archaea, and virus. **(B)** The structure and abundance of the top 10 phyla in both polyp and gallstone bile samples were analyzed using the intensity of identified microbial peptides. CHL, cholecystitis. **(C)** The structure and abundance of the top 10 microbial species in polyp and gallstone bile samples. **(D)** The taxa we identified were found in other Human body sites through data mining of public references. The details were deposited in Supplementary Table S2. **(E)** The tandem MS spectrum of peptide LYCEEVGWVICGIK identified from lepA protein (LEPA_BUCCC) of *Buchnera aphidicola*. **(F)** The Sankey plot shows the metataxonomic analysis of human bile samples. The dataset was retrieved from GenBank with the BioProject accession number PRJNA580086 (https://www.ncbi.nlm.nih.gov/bioproject/?term=PRJNA580086). In this project, 45 biliary bile samples were analyzed using 16S rRNA sequencing. We re-analyzed the dataset against kraken2-microbial database (https://lomanlab.github.io/mockcommunity/mc_databases.html) using kraken2 program (Lu et al., 2022). The red arrowhead represents the clade of *Buchnera aphidicola*, which has been detected in 44 out of 45 bile samples, comprising a total of 7,313 reads.

We successfully identified 711 distinct peptides that were specific to microorganisms. Utilizing these peptides, the LCA algorithm enabled the identification of 142 microbial taxa, each supported by a minimum of 3 distinct peptides (Supplementary Table S2). PLS-DA analysis using taxonomic data shared features between chronic and acute CHLs, while the polyp samples exhibited distinct characteristics from CHL (Figure 1D). We re-analyzed six 16S-rRNA gene sequencing datasets comprising a total of 479 bile samples and validated the presence of 86 of these bacterial phyla in bile samples, as shown in Supplementary Tables S2, S3. Additionally, through data mining of public references, we discovered that the majority of the taxa (139 out of 142) were found in various human body sites, except for bile (Supplementary Table S2), such as the gut/feces, scalp/skin, milk, oral tissues, vagina, etc. (Figure 2D). Among all the distinct peptides identified in our samples (a total of 23,033), microbial-specific peptides accounted for approximately 3% of the total (Figure 2A). This suggests that the microbial content in bile is relatively low in abundance.

Our analysis at the Phylum level revealed the presence of several bacterial phyla in bile samples, including Proteobacteria, Firmicutes, Actinobacteria, Bacteroidetes, Tenericutes, Chlorobi, Cyanobacteria, Chloroflexi, and Spirochaetes (Figure 2B; Supplementary Table S2). The identification of some phyla in the bile samples, like Chlorobi and Spirochaetes, is consistent with previous studies (Saltykova et al., 2016; Molinero et al., 2019). Among the fungal phyla, Ascomycota was the most abundant in bile samples. Euryarchaeota was the dominant Phylum among Archaea, and it has also been found in the human oral cavity (Dewhirst et al., 2010). In the human gut, Ascomycota is the most common fungal phylum, while Euryarchaeota is the most common archaeal Phylum (Xiong et al., 2022). We also identified the presence of the Genus Candida, belonging to the Phylum Ascomycota (Supplementary Table S2). *Candida* spp. is an infectious agent associated with CHL and has serious prognostic implications (Diebel et al., 1996). In terms of Viruses, Pisuviricota exhibited the highest abundance (Figure 2B; Supplementary Table S2). Pisuviricota is a Phylum of RNA viruses, including the Genus Cosavirus, which commonly causes acute gastroenteritis infections in pediatric patients (Razizadeh et al., 2021).

At the Class level, the most abundant Classes for Bacteria, Eukaryota, Archaea and viruses were Gammaproteobacteria, Saccharomycetes, Methanomicrobia, and Pisoniviricetes, respectively (Supplementary Figure S1A). Supplementary Figures S1B–D show the most abundant taxa at the Order, Family, and Genus levels. Among these taxa, Streptococcus and Clostridium are commonly found in gallstone samples and have been associated with acute CHL (Ploszaj et al., 2021; Shigemori et al., 2022). Notably, we identified that *Staphylococcus aureus* as the third most abundant Species in the bile samples (Figure 2C). Both Streptococcus and *S. aureus* are known to be β-glucuronidase-producing bacteria, and infections caused by these bacteria have been associated with the development of gallstone formation (Hancke and Marklein, 1983).

At the Species level, *Vibrio vulnificus* was identified as the most prevalent species, and its identification was supported by the metataxonomic analysis of bile datasets (Figure 2C; Supplementary Table S3). *V. vulnificus* is a pathogenic agent typically associated with exposure to seawater or consumption of contaminated seafood. Interestingly, its presence in human gallstone bile fluid suggests a potential link between *V. vulnificus* infection and gallstone formation (Zhao et al., 2015). While there is no specific evidence linking *Schizosaccharomyces pombe* to CHL, this yeast Species has been identified in the gut mycobiota of both healthy individuals and colorectal cancer patients (Chin et al., 2018). *Buchnera aphidicola* is typically recognized as an obligate endosymbiont of aphids, establishing a mutualistic relationship with their hosts. However, it is noteworthy that peptides of *B. aphidicola* were detected in all the bile samples in our study. This unexpected finding suggests the presence of *B. aphidicola* or its remnants in the bile samples, potentially indicating a broader ecological role or a possible association with gallstone disease beyond its traditional symbiotic relationship with aphids. Figure 2E displayed a representative tandem MS spectrum of the peptide LYCEEVGWVICGIK, which was identified from the lepA protein (LEPA_BUCCC) of *B. aphidicola*. Metataxonomic analysis of 16S rRNA gene sequencing datasets further validates its presence in human bile samples (Figure 2F). In fact, a study utilizing metagenomic shotgun sequencing has confirmed the presence of *B. aphidicola* in the human skin microbiome (Oh et al., 2016). The fifth Species we have identified is *Clostridium perfringens*, a bacterium that produces β-glucuronidase, and this particular bacterium has been linked to the formation of pigment gallstones (Skar et al., 1986). *Eremothecium gossypii* is a filamentous fungus known to be an infectious agent capable of producing riboflavin and glucosylceramide, and the accumulation of glucosylceramide it produces could contribute to cholelithiasis (Taddei et al., 2010). It is known that riboflavin plays a role in lipid metabolism and can influence cholesterol accumulation. *Neosartorya fumigata* (*Aspergillus fumigatus*), an opportunistic fungal pathogen and a major allergen, is the most common cause of invasive aspergillosis, particularly for immunocompromised individuals (O'Gorman et al., 2009).

### 3.3 Microbial markers of gallstone disease

**Figure 3:**
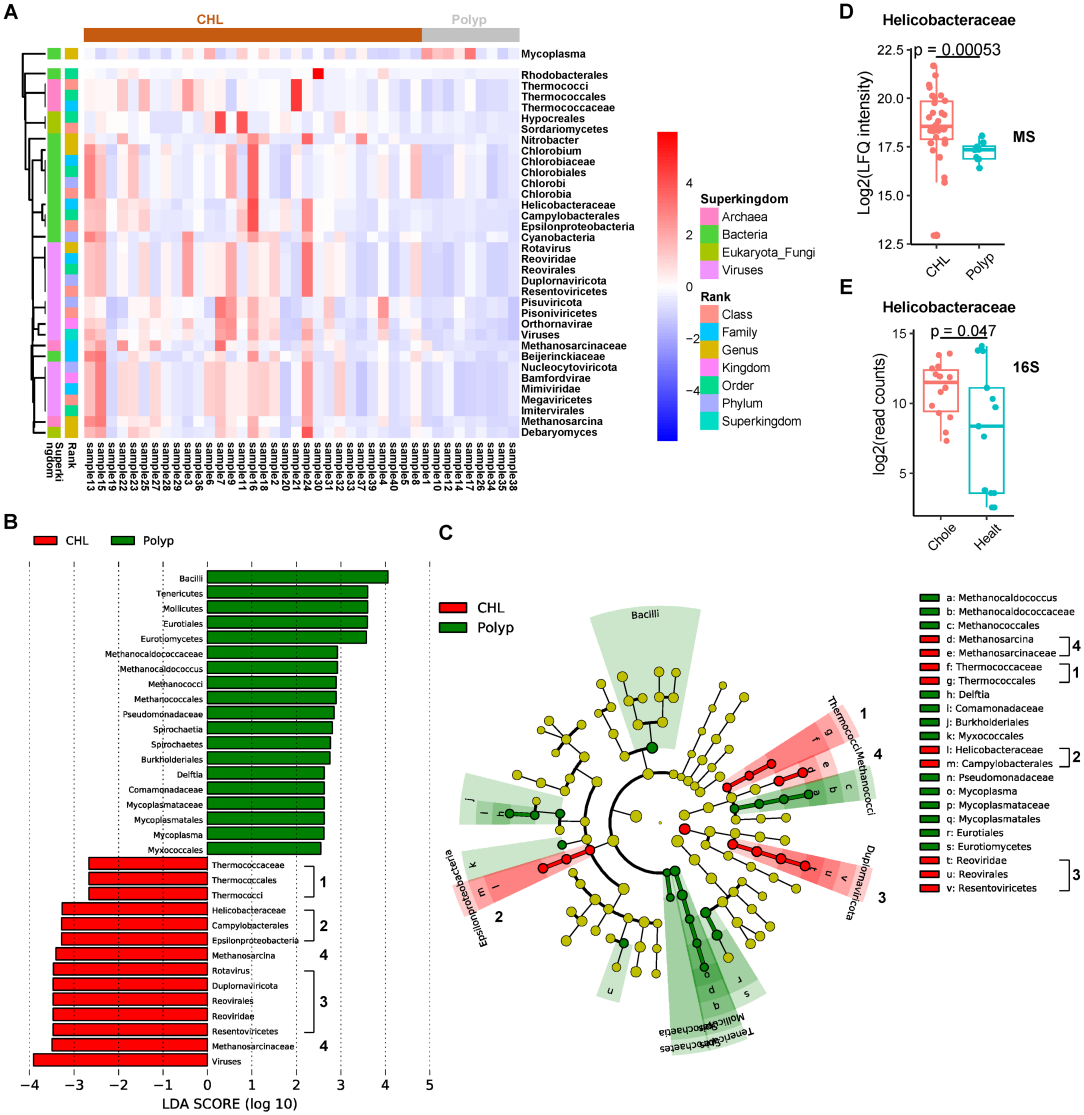
Taxon changes associated with gallstone formation determined by the microbial peptides. **(A)** Hierarchical cluster analysis of the microbial taxa significantly changed in gallstone bile samples (*n* = 31) comparing to polyp bile samples (*n* = 9). **(B,C)** Linear discriminant analysis Effect Size (LEfSe) analysis of microbial biomarkers of CHL (*n* = 31) and polyp (*n* = 9) using an LDA threshold of 2. The numbers indicate the major marker clades in CHL. **(D)** The abundance of Helicobacteraceae between CHL (*n* = 31) and polyp (*n* = 9) patients based on the proteomic data. The *p* values were calculated using Welch’s *t* test. MS, mass spectrometry. **(E)** The abundance of Helicobacteraceae between CHL (*n* = 14) and healthy (*n* = 13) patients based on the 16S rRNA data (dataset PRJNA439241). 16S, 16S rRNA dataset analysis. Chole, cholecystitis. Healt, healthy.

In the CHL group, we observed significant higher abundance of 34 phyla, including 3 fungi, 5 archaea, 12 bacteria, and 14 viruses, compared to the polyp group (Figure 3A; Supplementary Table S2). We identified 44 differential phyla between acute CHL and polyp bile samples, with 29 phyla common to the CHL and polyp comparison (Supplementary Figure S2A; Supplementary Table S2). Additionally, we found 27 differential phyla between chronic CHL and polyp bile samples, all of which were common to the CHL vs. polyp comparison (Supplementary Figure S2B; Supplementary Table S2). However, only four differential phyla were identified between acute and chronic CHL (Supplementary Figure S2C; Supplementary Table S2). To identify potential microbial biomarkers for CHL, we performed a LEfSe analysis. Interestingly, four clades were identified as microbial biomarkers in CHL (LDA score > 2), including the Thermococcaceae clade, Helicobacteraceae clade, Methanosarcina clade, and Reoviridae clade (Figures 3B,C). Among the Archaea we identified, Methanosarcina and the Thermococcaceae clade have been found in the human skin microbiome (Oh et al., 2016). In the bacterial phyla upregulated in CHL, Helicobacteraceae is a Family that belongs to the Order Campylobacterales and the Class Epsilonproteobacteria. Interestingly, a human cohort study reveals that Helicobacter infection is associated with an increased risk of gallstone disease (Cen et al., 2023). Our analysis detected Helicobacteraceae peptides in 39 out of 40 bile samples (Figure 3D). Furthermore, upon re-analyzing the dataset PRJNA439241, we detected 16S rRNA reads of Helicobacteraceae in all 27 bile samples (Figure 3E; Supplementary Tables S2, S3). Within the Chlorobium clade, Chlorobia, Chlorobiaceae, Chlorobiales, and Chlorobium have been identified in human skin microbiome (Oh et al., 2016), while Chlorobi (Phylum) was identified in bile (Molinero et al., 2019). The presence of Beijerinckiaceae in bile was confirmed by reanalysis of 16S rRNA datasets of bile (4 out of 6) (Supplementary Tables S2, S3), and it was also identified in human scalp (Futatsuya et al., 2020). Cyanobacteria was widely distributed in the human body, not only in gallstone bile (Wu et al., 2013), but also in colon (Stearns et al., 2011), scalp (Futatsuya et al., 2020), and stool (Morales et al., 2022). Notably, Cyanobacteria, well-known for its biofilm production capability, may contribute to gallstone formation within the bile environment (Maeda et al., 2021). Rhodobacterales was found to be a bile biomarker in a study analyzing bile and gallstone samples (Hu et al., 2023). Mycoplasma was the only bacteria downregulated in CHL. As a human pathogen, Mycoplasma has been detected in various human locations, including bile, subgingival and buccal mucosa (Griffen et al., 2012; Kraal et al., 2014; Chen et al., 2019). Its presence in bile was further validated by reanalysis of bile 16S rRNA datasets (Supplementary Tables S2, S3). We also revealed several differential virus phyla in CHL samples. Bamfordvirae, Nucleocytoviricota, Megaviricetes, Imitervirales, and Mimiviridae were identified based on the same set of peptides (Supplementary Table S2). Nucleocytoviricota, although not identified in our analysis of 16S rRNA datasets, has been found in the human ocular surface (Shao et al., 2023), while Mimiviridae or Minivirus has been identified in skin microbiome (Oh et al., 2016) or pulmonary samples (Saadi et al., 2013). The Reoviridae clade, as well as Pisuviricota (Pisoniviricetes), belongs to the Kindom Orthornavirae. Reoviridae (Duplornaviricota, Reovirales, Resentoviricetes, Rotavirus) has been commonly implicated in biliary atresia, a childhood disease (Averbukh and Wu, 2018). Among the fungal taxa, Debaryomyces has been previously identified in the human digestive tract and associated with Crohn’s disease (Olaisen et al., 2022), while Sordariomycetes (Hypocreales) was found in skin microbiome (Oh et al., 2016).

### 3.4 Identification of microbial protein groups

**Figure 4:**
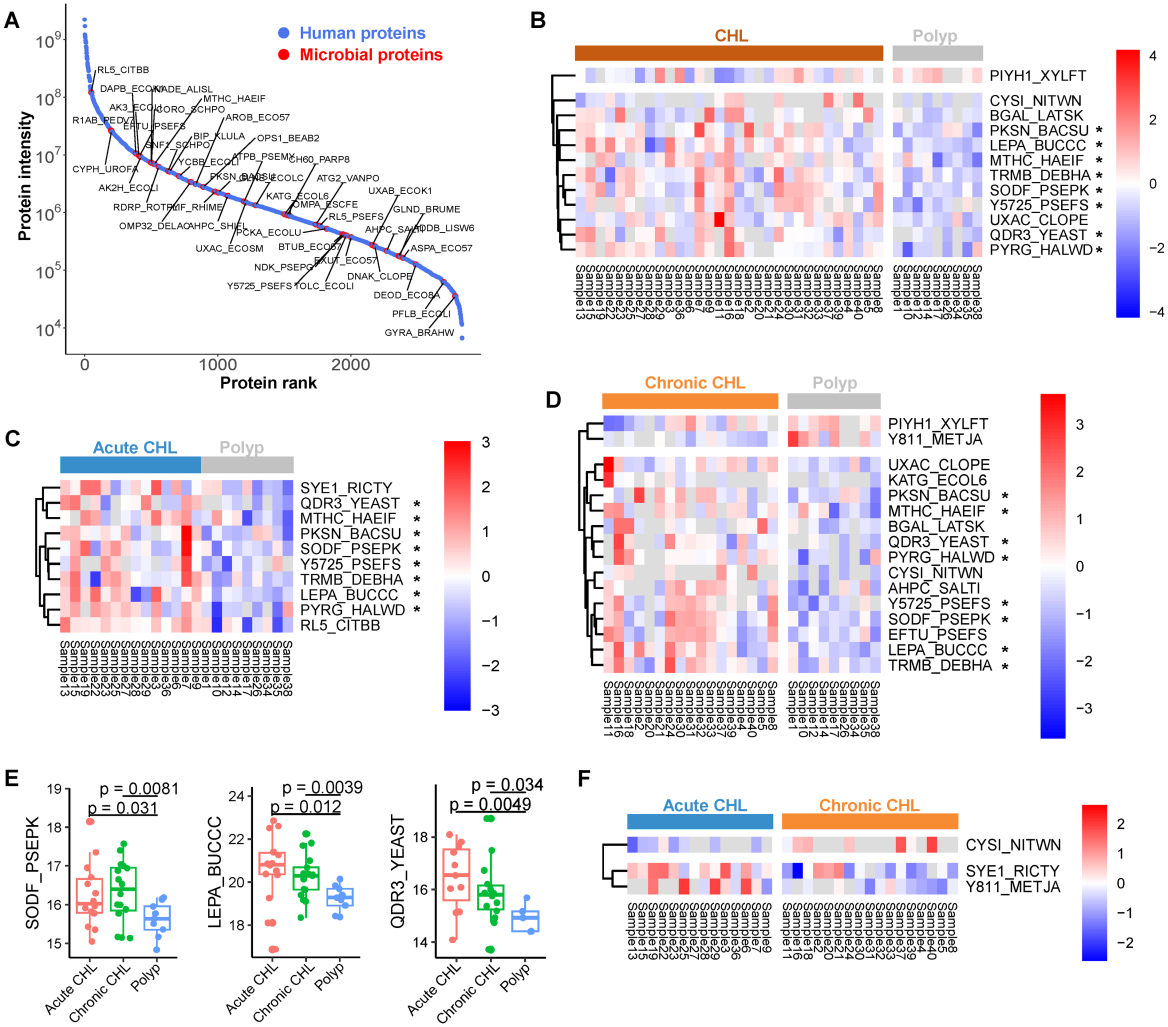
The microbial proteins identified from gallstone and polyp bile samples. **(A)** Abundance rank of identified proteins. The intensity of each protein was determined by summing up the LFQ values across all samples. Microbial proteins were highlighted. **(B)** The abundance of differential microbial proteins between CHL (*n* = 31) and polyp (*n* = 9) patients. **(C)** The differential microbial proteins between acute CHL (*n* = 14) and polyp (*n* = 9) patients. **(D)** The differential microbial proteins between chronic CHL (*n* = 17) and polyp (*n* = 9) patients. **(E)** Three microbial proteins upregulated in CHL compared to polyp. *p* values were calculated using Welch’s *t* test. **(F)** The differential microbial proteins between acute CHL (*n* = 14) and chronic CHL (*n* = 17) patients.

We identified a total of 87 microbial proteins (Figure 4A; Supplementary Table S4), with 47 (54%) of them associated with the Phylum Proteobacteria. It is noteworthy that the ability of bacteria to cause biliary infection plays a crucial role in gallstone formation by promoting the production of biofilms, also known as slime or glycocalyx, which facilitates bacterial colonization and solidification of gallstones. Among the identified microbial proteins, several are known to be involved in biofilm formation. We observed a gradual upregulation of quinidine resistance protein 3 (QDR3), a quinidine drug resistance protein from Saccharomyces, from chronic CHL to acute CHL when compared to polyp samples (Figures 4B–E). This protein is instrumental in biofilm formation and enhances the virulence of fungal pathogens (Shah et al., 2014). Similarly, ompA, an outer membrane protein found in *Escherichia. coli*, is crucial for the complete formation of certain bacterial biofilms in a bile environment (Tsai et al., 2020). The nucleoside diphosphate kinase (ndk) protein in Pseudomonas not only influences cytotoxicity and host pathogenicity (Yu et al., 2016), but also plays a role in biofilm production (Sikarwar et al., 2022). Pseudomonas ndk is a critical host-responsive gene that regulates bacterial virulence during infection, as its expression was downregulated during acute pneumonia (Yu et al., 2016). The phosphate-binding protein pstS, identified in Pasteurella (Supplementary Table S4), has been shown to play an important role in biofilm formation in its homolog in Pseudomonas (Neznansky et al., 2014). N-acetylneuraminate pyruvate-lyase (nanA) in Clostridium is a sialic acid aldolase, and sialic acid is associated with bacterial pathogenicity. The ortholog of this enzyme in other bacteria has been demonstrated to participate in biofilm formation (Di Pasquale et al., 2016). Formate acetyltransferase 1 (pfIB) in *E. coli* also contributes to biofilm formation by regulating acytyl-CoA concentrations (Mugabi et al., 2012). Similarly, the chaperone protein dnaK in Clostridium enhances biofilm formation (Jain et al., 2017).

We have identified several microbial proteins that enhance bacterial virulence and gallstone formation by counteracting host oxidative stress. One of these proteins is superoxide dismutase (SOD), which acts as the primary scavenger of host oxidative superoxide anion. We found that Pseudomonas iron-cofactored SOD [Fe] (sodB/SODF_PSEPK) was upregulated in both acute and chronic CHL compared to polyp samples (Figures 4B–E). *Pseudomonas aeruginosa* has been revealed as the dominant agent associated with gallstone disease (Peng et al., 2015) and acute CHL (Sakurai et al., 1992). sodB plays a vital role in enabling Pseudomonas to survive against host oxidative stress (Cavinato et al., 2020). KatG, a catalase-peroxidase, plays a crucial role in protecting the biofilm of certain bacteria by neutralizing external oxidative stress, thereby contributing to biofilm growth on cholesterol gallstones (von Beek et al., 2019). Rubrerythrin (rbr) in Clostridium functions as a scavenger of oxygen radicals, aiding in the protection of bacteria against oxidative stress (Jean et al., 2004). The serine protease htrA, implicated in the virulence of many pathogens including Staphylococcus (Rigoulay et al., 2005), contributes to pathogenicity by regulating the expression of essential virulence factors and stress proteins. Salmonella has been associated with gallstone development due to its ability to form biofilms on the cholesterol-coated surface of gallstone (Gonzalez et al., 2019). We identified a protein in Salmonella, the alkyl hydroperoxide reductase subunit C (ahpC). This peroxiredoxin reduces organic peroxide, protecting Salmonella from host oxidative stress and enhancing its virulence (Hebrard et al., 2009).

We also identified microbial proteins that contribute to bacteria virulence and potential play a role in gallstone formation. Similar to QDR3, Buchnera lepA exhibited a gradual upregulation from chronic to acute CHL compared to polyp samples (Figures 4B–E). LepA also known as elongation factor 4 (EF-4), is a GTP-binding protein that acts a virulence factor in several bacteria (Kida et al., 2011). The RtxA gene, which encodes heat-stable enterotoxin, has been identified as an essential virulence factor of *Vibrio vulnificus* (Lee et al., 2007). The pckA gene, which encodes phosphoenolpyruvate carboxykinase (PEPCK), acts as a pathogenic factor for virulent bacteria (Liu et al., 2005). Tuf, the elongation factor Tu (EFTU) in Pseudomonas, was identified by 13 unique or razor peptides in bile (Supplementary Table S4). Tuf can serve as a virulence factor for Pseudomonas, enabling it to evade human complement attack by binding to the human complement regulator Factor H and plasminogen (Kunert et al., 2007). Pseudomonas, *E. coli* and Staphylococcus are biofilm-forming bacteria that have been identified in bile stone samples (Tajeddin et al., 2016). The capability of Pseudomonas to form biofilms on the surface of implanted gallstones has been confirmed in animal models (Sung et al., 1991). TpiA, a key metabolic triosephosphate isomerase of pathogenic bacteria, affects virulence, antibiotic resistance, and fitness within the host (Paterson et al., 2009; Xia et al., 2020), and its upregulation in CHL may indicate the adaptation of invading bacteria to the altered host environment.

The identification of microbial proteins in our study encompasses transporters that counteract bile salt stress or drug attack, thereby facilitating microbial survival in bile environment. One of these proteins is the outer membrane protein TolC, which functions as a crucial pump in *E. coli,* enabling the efflux of various substances like bile salts (Rosner and Martin, 2009). Additionally, QDR3, a multidrug transporter in yeast, has been identified in bile samples.

In comparing acute and chronic CHL, we observed differential expression of three microbial proteins: cysI (CYSI_NITWN), gltX1 (SYE1_RICTY), and MJ0811 (Y811_METJA) (Figure 4F). These findings suggest that there is only a minimal difference in microbial expression between the two sample types.

### 3.5 Enhanced host inflammatory in cholelithiasis

**Figure 5:**
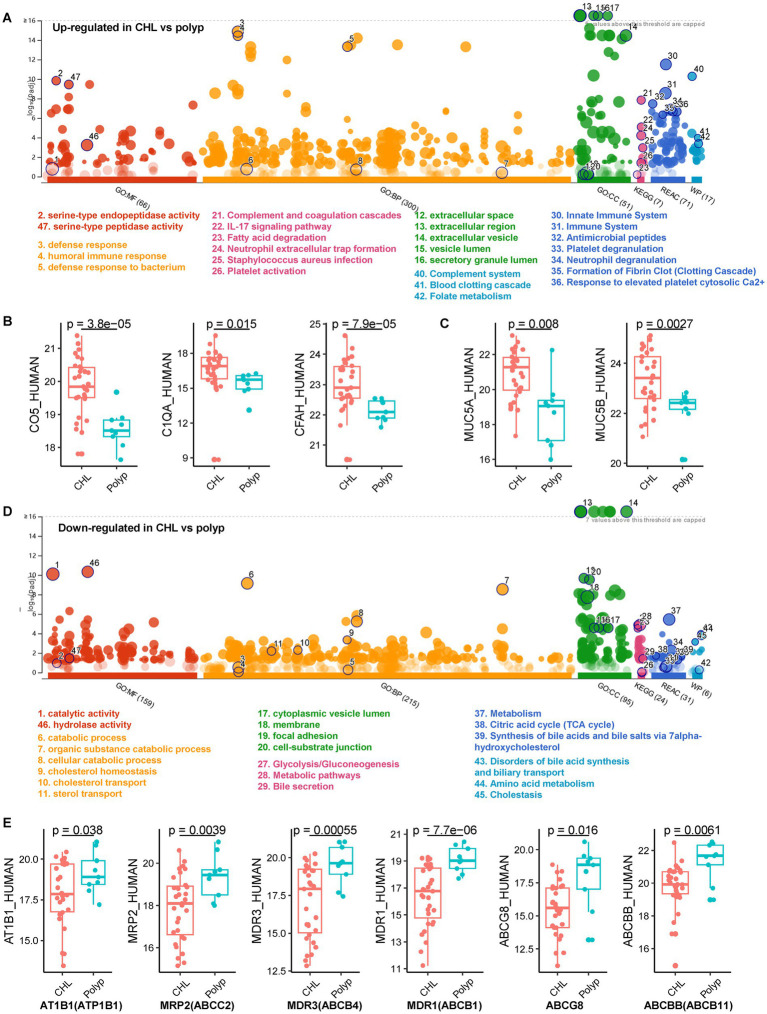
The host proteins identified from gallstone and polyp bile samples. **(A)** Gene ontology (GO) enrichment was performed on the upregulated human proteins in CHL compared to polyp using the g:Profiler tool (https://biit.cs.ut.ee/gprofiler/gost) (Raudvere et al., 2019). **(B)** Differential expression of complement proteins between CHL and polyp samples. *p* values were calculated using unpaired Welch’s *t* test. **(C)** Two mucin proteins were upregulated in CHL compared to polyp. **(D)** GO enrichment of the downregulated human proteins in CHL compared to polyp. GO_MF, Gene ontology of molecular function; GO_CC, cellular component; GO_BP biological process; REAC, Reactome; WP, WikiPathways. **(E)** Proteins associated with bile secretion were downregulated in CHL compared to polyp.

We identified a total of 2,749 host proteins across all bile samples (Supplementary Table S5), including 67 upregulated and 188 downregulated proteins in the cholelithiasis group (*n* = 31) compared to the polyp group (*n* = 9) (CHL_vs_polyp), with an absolute log2-fold change ≥1 and a *p* value < 0.05 (Supplementary Table S5). We then performed a functional enrichment analysis using g:Profiler (Raudvere et al., 2019). The analysis revealed that the upregulated proteins in CHL were significantly enriched in serine-type endopeptidase activity (*p*_adjusted_ = 7.0E-11), heparin binding (*p*_adjusted_ = 1.3E-07), and complement binding (*p*_adjusted_ = 0.0009) (Supplementary Table S6; Figure 5A). Moreover, the GO Biological Process (GO_BP) terms in CHL were primarily associated with various immune response processes, including humoral immune response (*p*_adjusted_ = 4.0E-19) and defense response (*p*_adjusted_ = 2.2E-19) (Supplementary Table S6). These proteins comprised immunoglobulins (Igs), C4BPA, RNASE3, CTSG, AZU1, ELANE, PRTN3, S100A9, DEFA3, FGA, and others. Additionally, the presence of complement-activation-associated proteins like C4BPA, C1QA, C5, CFP, CD5L, IGHG2, A2M, and CFH, was increased in CHL bile (Figure 5B). KEGG analysis indicated that the upregulated proteins in CHL were predominantly involved in pathways such as complement and coagulation cascades (*p*_adjusted_ = 1.4E-08) and neutrophil extracellular trap formation (*p*_adjusted_ = 6.1E-05). Consistently, Reactome (REAC) analysis revealed that CHL-upregulated proteins were significantly enriched in the innate immune system (*p*_adjusted_ = 3.1E-12), complement cascade (*p*_adjusted_ = 6.6E-07) and antimicrobial peptides (*p*_adjusted_ = 3.5E-08). Furthermore, the WikiPathways (WP) analysis indicated an overrepresentation of the complement system (*p*_adjusted_ = 5.2E-11) among the upregulated proteins in CHL (Supplementary Table S6; Figure 5A). In the GO cellular component (GO_CC) analysis, the upregulated proteins were primarily enriched in the extracellular space (*p*_adjusted_ = 3.0E-23), while the downregulated proteins showed enrichment in both the extracellular space (*p*_adjusted_ = 1.8E-44) and the cytoplasm (*p*_adjusted_ = 3.7E-14).

We identified 74 increased and 362 decreased host proteins in acute CHL (*n* = 14) compared to polyp (*n* = 9) (acute_vs_polyp) (Supplementary Figure S3; Supplementary Table S5). Similarly, we identified 51 upregulated and 95 downregulated host proteins in chronic CHL (*n* = 17) compared to polyp (*n* = 9) (chronic_vs_polyp) (Supplementary Table S5; Supplementary Figure S4). The enrichment analyses of these differential proteins yielded results to the CHL vs. polyp comparison, suggesting a potential role of complement activation in the cholelithiasis process. To validate the expression of selected complement proteins, we conducted a dot plot analysis. As shown in Supplementary Figure S5, consistent with the proteomic quantitation results, the expression of CFI remained constant between polyp and gallstone bile samples, while C5 was found to be increased in gallstone bile samples.

These findings indicate the presence of an inflammatory feature in CHL and the hyperactivation of both adaptive and innate immune pathways in the host bile environment.

### 3.6 Increased host defense against bacterial infection

Five GO:BP terms exhibited specific enrichment in proteins upregulated in CHL, including defense response to bacterium (*p*_adjusted_ = 6.8E-14) response to bacterium (*p*_adjusted_ = 2.4E-11), antimicrobial humoral response (*p*_adjusted_ = 2.1E-13), antibacterial humoral response (*p*_adjusted_ = 9.5E-10), and defense response to fungus (*p*_adjusted_ = 5.1E-10) (Supplementary Table S6). Notably, 17 human proteins were upregulated in the defense response to bacterium, including C4BPA, RNASE3, CTSG, MMP9, RETN, AZU1, EPX, MPO, ELANE, SERPINB4, PRTN3, LCN2, CRP, S100A9, S100A8, HNRNPA0, DEFA3, APCS, FGA, NT5C3A, HRG, F12, LTF, FGB, FGG, RTN4R, C1QA, C5, CFP, TTN, IGHG2, A2M, TF, PPP1CB, and CFH. Furthermore, the upregulated proteins in CHL demonstrated enrichment in *Staphylococcus aureus* infection (*p*_adjusted_ = 0.001) based on the KEGG analysis, and in antimicrobial peptides (*p*_adjusted_ = 3.5E-8) according to the REAC analysis. These results strongly suggest a crucial role for the antimicrobial immune response in CHL.

### 3.7 Coagulation and other pathways activated in CHL

We observed a significant upregulation of proteins in CHL compared to polyp, specifically those involved in blood coagulation and fibrin clot formation (*p*_adjusted_ = 7.1E-08, GO_BP), formation of fibrin clot (clotting cascade) (*p*_adjusted_ = 4.5E-07, REAC), common pathway of fibrin clot formation (*p*_adjusted_ = 2.9E-05, REAC), and blood clotting cascade (*p*_adjusted_ = 0.0001, WP) (Supplementary Table S6; Figure 5A). Similar enrichments were observed in the differential proteins of acute_vs_polyp and chronic_vs_polyp. Notably, the proteins involved in these processes were FGA, F12, FGB, FGG, FN1, PRTN3, and A2M. Furthermore, we found an overrepresentation of the folate metabolism pathway in CHL (*p*_adjusted_ = 4.1E-04), which includes components MPO, CRP, FGA, FGB, and FGG. Additionally, the interleukin-17 (IL-17) signaling pathway (*p*_adjusted_ = 8.2E-06) was enriched in the upregulated protein in CHL, including MMP9, LCN2, S100A9, S100A8, MUC5AC, MUC5B, and MMP1. It is worth mentioning that among the seven mucin (MUC) proteins, MUC5A and MUC5B exhibited a significant increase in gallstone bile compared to polyp bile (Figure 5C). These findings are consistent with previous findings, which suggest that high-molecular-weight glycoproteins, such as intraluminal mucin deposition, may contribute to gallstone formation (Yoo et al., 2016).

### 3.8 Host catabolic metabolism activity was suppressed in gallstone bile

In gallstone bile (*n* = 31) compared to polyp (*n* = 9), a total of 188 human proteins exhibited downregulation. These proteins showed enrichment in GO_MF terms related to catalytic activity (299 proteins, 53%) (*p*_adjusted_ = 2.7E-14) and hydrolase activity (*p*_adjusted_ = 4.3E-14) (Supplementary Table S6; Figure 5D). GO_BP analysis revealed that many of the downregulated host proteins were associated with “metabolic” or catabolic,” such as catabolic process (*p*_adjusted_ = 1.6E-16, 71 proteins, 37.8%), small molecule metabolic process (*p*_adjusted_ = 1.1E-11), and organic substance catabolic process (*p*_adjusted_ = 2.0E-14). This finding indicates that gallstone disease significantly suppresses metabolic activity, particularly catabolic metabolism. Moreover, this conclusion was further supported by KEGG, REAC, and WP analyses (Figure 5D). Additionally, GO_CC analysis revealed that the downregulated proteins were not only enriched in extracellular/secreted proteins but also in cytoplasmic proteins, which aligns with the fact that many metabolism-associated proteins are primarily located in the cytoplasm.

### 3.9 Bile secretion and transportation were impaired in gallstone formation

The proteins downregulated in CHL, in comparison to polyps, demonstrated enrichment in the KEGG pathway of bile secretion (*p*_adjusted_ = 0.039), which included ATP1B1, ABCC2, ABCB11, ABCB4, ABCB1, and ABCG8 (Figures 5D,E; Supplementary Table S6). Furthermore, we observed a significant downregulation of host proteins related to disorders of bile acid synthesis and biliary transport (WP:WP5176) in gallstone bile (*p*_adjusted_ = 0.0001). Notable proteins in this category were AKR1D1, ABCC2, HSD17B4, ABCB11, ABCB4, and ATP8B1 (Supplementary Table S6). Additionally, the downregulated proteins in gallstone bile showed enrichment in bile acid and bile salt transport (*p*_adjusted_ = 0.004), which included AKR1C1, ABCC2, ABCB11, and ATP8B1. These findings indicate a disruption in bile secretion in CHL compared to polyp, potentially leading to bile supersaturation and the formation of cholesterol-type gallstones.

### 3.10 Catabolic processes were further suppressed in acute CHL compared to chronic CHL

We identified 8 upregulated and 95 downregulated proteins in acute CHL (*n* = 14) compared to chronic CHL (*n* = 17) (Supplementary Figure S6A; Supplementary Table S6). No enrichment was observed among the upregulated proteins (Supplementary Figure S6B). However, the downregulated proteins exhibited enrichment in metabolic pathways, particularly the catabolic process (*p*_adjusted_ = 0.0011), cellular catabolic process (*p*_adjusted_ = 0.0006), and cytoplasmic translation (*p*_adjusted_ = 3.2E-07) (Supplementary Figure S6C; Supplementary Table S6). These findings indicate a further suppression of catabolic activities in acute CHL compared to chronic CHL.

## 4 Discussion

In this study, we performed an integrated proteomic and metaproteomic analysis of human bile samples obtained from individuals with gallstone and polyp disease. Utilizing LCA algorithm, we performed a taxonomic analysis of the microbiome based on 711 identified microbial peptides, and identified 142 microbial taxa. The lower abundance of microbial proteins in bile contributed to the relatively small identification rate of microbial peptides compared to human peptides. While this may affect the composition analysis of microbial taxa, the lack of appropriate enrichment methods for microbial peptides limits the improvement in identification. To validate the presence of these taxa in bile samples, we re-analyzed six 16S rRNA gene sequencing datasets, confirming the presence of 60% of the identified taxa. Furthermore, publicly available references demonstrated that the majority of these taxa were also found in different body sites of humans. These findings provide robust evidence for the reliability of our metaproteome results and underscore the potential of metaproteomics in detecting previously undiscovered microbial entities that may have been missed by DNA sequencing techniques. Notably, our metaproteomic analysis uncovered the presence of microbes, such as *B. aphidicola*, an endosymbiont commonly found in aphids, which were previously not considered to have a relationship with humans. The predominant bacterial phyla identified in gallstone bile through metaproteomic analysis were Proteobacteria, Firmicutes, Actinobacteria, and Bacteroidetes, displaying similarities to the composition revealed by 16S rRNA sequencing, albeit with differences in taxonomic order (Molinero et al., 2019). Several factors, including the distinct nature of nucleotides and proteins, sample variation, and potential limitations in data acquisition, may contribute to this discrepancy.

Gallstones can be categorized into three major types: cholesterol stones, brown pigment stones, and black pigment stones (Wang Y. et al., 2018). In our study, we identified taxa known to produce β-glucuronidase, such as Streptococcus, *S. aureus*, and Clostridium. Bacteria-derived β-glucuronidase hydrolyzes bilirubin conjugates in gallbladder bile, leading to the release of free bilirubin and glucuronic acid, which preciptates as insoluble salts with ionized calcium. This process is considered the primary mechanism underlying the formation of brown pigment gallstones (Wang Y. et al., 2018). Through LEfSe analysis, we identified marker taxa in gallstone disease bile, including the Helicobacteraceae clade, which was present in almost all bile samples. Helicobacter infection is commonly considered as a potential risk factor for gallstone disease (Cen et al., 2023). However, experiments using animal models of *H. pylori* infection to study cholesterol gallstone formation have not provided conclusive evidence (Maurer et al., 2006). Bacterial biofilm plays a significant role in bacteria colonization and gallstone solidification, serving as an additional pathway for gallstone formation (Wang Y. et al., 2018). Bacteria with slime activity, such as Salmonella, Enterococcus, and Neisseria, have been observed to promote gallstone solidification (Sharma et al., 2020). Our identification of bacteria responsible for biofilm formation includes Cyanobacteria (Maeda et al., 2021), Pseudomonas (Sikarwar et al., 2022), *E. coli* (Tsai et al., 2020), and Clostridium (Jain et al., 2017).

In our analysis, we successfully identified 87 microbial proteins that shed light on the molecular mechanisms underlying gallstone formation. Firstly, we discovered several microbial proteins involved in biofilm production, such as ndk, QDR3, ompA, pstS, dnaK, nanA, and pflB. These proteins originated from microbes such as Pseudomonas, Saccharomyces, *E. coli*, Pasteurella, or Clostridium. Secondly, we identified microbial proteins that enhance bacterial virulence and contribute to gallstone formation by counteracting host oxidative stress. These proteins include sodB, KatG, rbr, HtrA, and ahpC. Gallstone patients exhibit high levels of oxidative stress in the gallbladder mucosa (Geetha, 2002). Oxidative stress facilitates the rapid oxidation of bilirubin and free radical polymerization, which play a crucial role in gallstone nucleation and formation (Sanikidze and Chikvaidze, 2016). Thirdly, we discovered several microbial proteins that contribute to bacterial virulence and potentially induce gallstone formation, such as Buchnera lepA, RtxA gene, pckA gene, Tuf, and TpiA. These proteins have diverse roles in bacterial virulence, toxins production, immune evasion, biofilm formation, and adaptation to host environment changes, which have been addressed through gene expression studies, bile stone samples analysis, and animal models. Lastly, we identified microbial proteins, including transporters like TolC and QDR3, that enable microbial survival in the bile environment by counteracting bile salt stress or drug attacks.

We identified a total of 2,749 human proteins, among which 67 were significantly upregulated and 188 were downregulated in gallstone bile compared to gallstone-free bile. The host protein profile revealed an enhanced immune or inflammatory response associated with gallstone and CHL diseases. The upregulated human bile proteins were predominantly enriched in immune or inflammation-related biological processes or pathways, indicating the inherent inflammatory state of CHL. Notably, the complement system and antimicrobial peptides were overrepresented in CHL bile, suggesting excessive activation of the innate immune system. This finding is consistent with gene RNA-seq analysis conducted on both gallstone and polyp patients (Li et al., 2014). The increased levels of these enzymes may indicate a pancreatic leak in the context of CHL inflammation and pancreatitis. Additionally, we observed that the increased host proteins in gallstone bile are associated with resistance against microbial infection. Gallbladder S100A9 and S100A8 have been implicated in proinflammatory functions in CHL, particularly during the chronic stage (Szmyt et al., 2013).

In addition to the previously mentioned pathways, several other crucial pathways were found to be overrepresented in CHL, including blood coagulation, folate metabolism, and the IL-17 pathway. In cases of inflammatory gallstone disease, bleeding and the formation of fibrin clots may occur in the biliary tract (Chang et al., 2020). WP analysis indicated a significant alteration in folate metabolism, potentially implicating its involvement in gallstone disease (Worthington et al., 2004). Notably, among the proteins associated with folate metabolism, MPO exhibited a significant increase during gallstone development and mucus layer thickening in a mouse model (39). Furthermore, C-reactive protein (CRP), a marker of chronic inflammation, showed a significant association with a higher risk of gallstone disease in population studies (Barahona Ponce et al., 2021). The activation of the IL-17 pathway, which leads to inflammation and plays a significant role in cholestasis potentially induced by gallstone disease, was also observed (Tedesco et al., 2018). IL-17 is a proinflammatory cytokine, and CD4-positive Th17 cells that secrete IL-17 have been implicated in the chronic inflammation of bile ducts (Harada and Nakanuma, 2010).

In our analysis of downregulated proteins, we observed a significant suppression of metabolic activities, particularly catabolic metabolism, and transport activities in gallstone bile. Furthermore, the downregulated proteins were enriched in pathways related to bile secretion, bile acid synthesis, and biliary transportation. These changes contribute to the gradual accumulation of cholesterol and subsequent deposition of gallstones. We also identified host proteins involved in the formation of cholesterol-type gallstones. Notably, proteins associated with cholesterol transportation exhibited significant downregulation in gallstone bile. ABCG5/ABCG8, which function as hepatic and intestinal cholesterol transporters, form dimers to export sterols out of enterocytes and hepatocytes (Rebholz et al., 2018). Their downregulation leads to decreased phosphatidylcholine levels and increased cholesterol concentration, thereby promoting gallstone formation. Similarly, downregulation of ABCB11 and ABCB4, which act as bile salt exporters and phosphatidylcholine translocators, respectively, reduce cholesterol solubility (Chan and Vandeberg, 2012; Wang Y. et al., 2018). The downregulation of AKR1C4 and AKR1D1, which are involved in the synthesis of bile acids and bile salts that enhance cholesterol solubility, may promote gallstone formation (Wang Y. et al., 2018). Additionally, downregulation of NPC1L1, ABCG5, ABCG8, and LDLR, which are involved in intestinal and hepatobiliary cholesterol absorption, reduces cholesterol reabsorption from bile. This leads to biliary cholesterol supersaturation and subsequent gallstone deposition (Mokhtar et al., 2022).

## 5 Conclusion

In this study, we conducted a proteomic and metaproteomic analysis to explore the microbial features of gallstone and gallstone-free bile samples. We identified microbes and microbial factors involved in β-glucuronidase activity, biofilm formation, virulence, and anti-oxidative stress, which may contribute to gallstone development. Gallstone bile, or cholelithiasis bile, exhibited an enhanced inflammatory molecular profile, including molecules associated with the innate immune system’s response to microbial infections. Overrepresentation of pathways related to blood coagulation, folate metabolism, and IL-17 was observed in gallstone bile. Conversely, host metabolic activities, particularly catabolic metabolism and transport activities, were significantly reduced in gallstone bile. Additionally, we found that acute cholelithiasis bile exhibited more pronounced impairment in metabolic activities compared to chronic cholelithiasis bile. In conclusion, our analysis provide insights into the dysbiosis of resident microbes and the molecular interplay between the microbiome and host, contributing to the understanding of gallstone formation.

## Data availability statement

The datasets presented in this study can be found in online repositories. The names of the repository/repositories and accession number(s) can be found in the article/Supplementary material.

## Author contributions

X-TY: Data curation, Formal analysis, Investigation, Visualization, Writing – review & editing. JW: Formal analysis, Methodology, Validation, Investigation, Writing – review & editing. Y-HJ: Formal analysis, Methodology, Investigation, Writing – review & editing. LZ: Formal analysis, Methodology, Investigation, Writing – review & editing. LD: Data curation, Resources, Investigation, Writing – review & editing. JL: Conceptualization, Resources, Data curation, Writing – review & editing. FL: Conceptualization, Formal analysis, Funding acquisition, Project administration, Resources, Supervision, Visualization, Writing – original draft, Writing – review & editing, Data curation.

## Ethics statement

The studies involving humans were approved by Institutional Research Ethics Committee of the Institutes of Biomedical Sciences, Fudan University. The studies were conducted in accordance with the local legislation and institutional requirements. The participants provided their written informed consent to participate in this study.

## Footnotes

^1^https://proteowizard.sourceforge.io/

^2^https://www.uniprot.org/

^3^https://biit.cs.ut.ee/gprofiler/gost

^4^http://huttenhower.sph.harvard.edu/galaxy/

^5^https://ccb.jhu.edu/software/kraken2/

^6^https://fbreitwieser.shinyapps.io/pavian/

^7^http://proteomecentral.proteomexchange.org

^8^https://iprox.org/
