# Supplementary material for: Insight into the mechanism of gallstone disease by proteomic and metaproteomic characterization of human bile
Source: Front Microbiol. 2023 Dec 4;14:1276951. doi: 10.3389/fmicb.2023.1276951 (PMC10726133; doi:10.3389/fmicb.2023.1276951)
Supplement: Supplementary file 7 [file Data_Sheet_1.DOCX]

Supplementary Figures


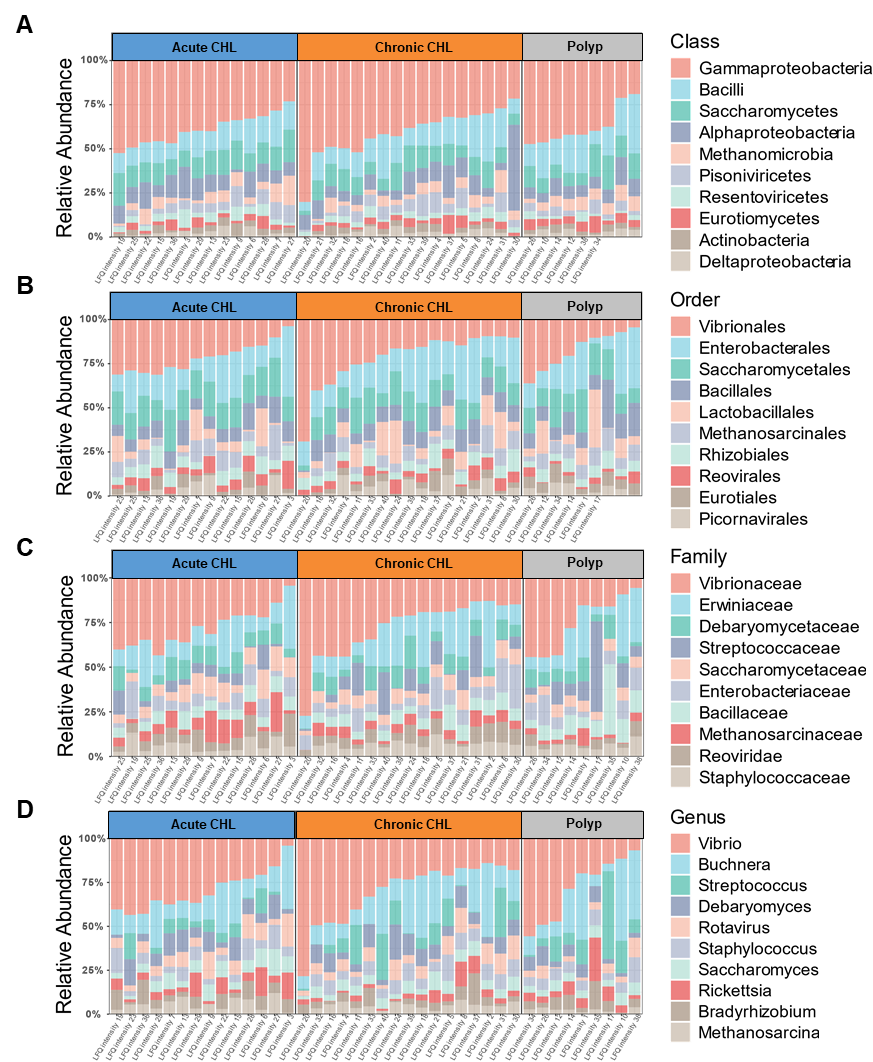


**Supplementary Figure 1.** **Taxon abundance between gallstone and polyp bile samples determined by the microbial peptides**. (**A**). Structure and abundance of the top ten Classes in polyp and gallstone bile samples (acute cholecystitis (CHL) and chronic cholecystitis) as a function of peptide intensity of the identified microbial peptides. (**B**). Structure and abundance of the top ten Orders in gallstone-free and gallstone bile samples as a function of peptide intensity of the identified microbial peptides. (**C**). Structure and abundance of the top ten Families in polyp and gallstone bile samples as a function of peptide intensity of the identified microbial peptides. (**D**). Structure and abundance of the top ten Genera in gallstone-free and gallstone bile samples as a function of peptide intensity of the identified microbial peptides.


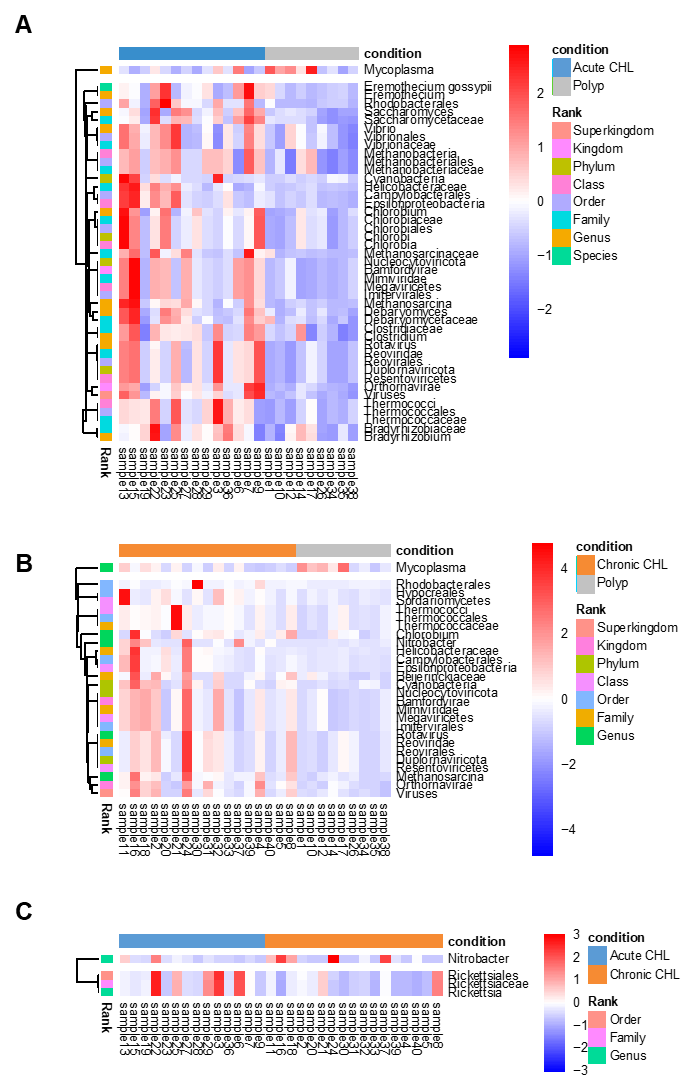


**Supplementary Figure 2.** **Hierarchical clustering analysis of differential microbial taxa between gallstone and polyp bile samples.** (**A**) Microbial taxa significantly changed in acute CHL bile samples (n=14) comparing to polyp bile samples (n=9). (**B**). Microbial taxa significantly changed in chronic CHL bile samples (n=17) comparing to polyp bile samples (n=9). (**C**) Microbial taxa significantly changed in acute CHL (n=14) and chronic CHL (n=17).


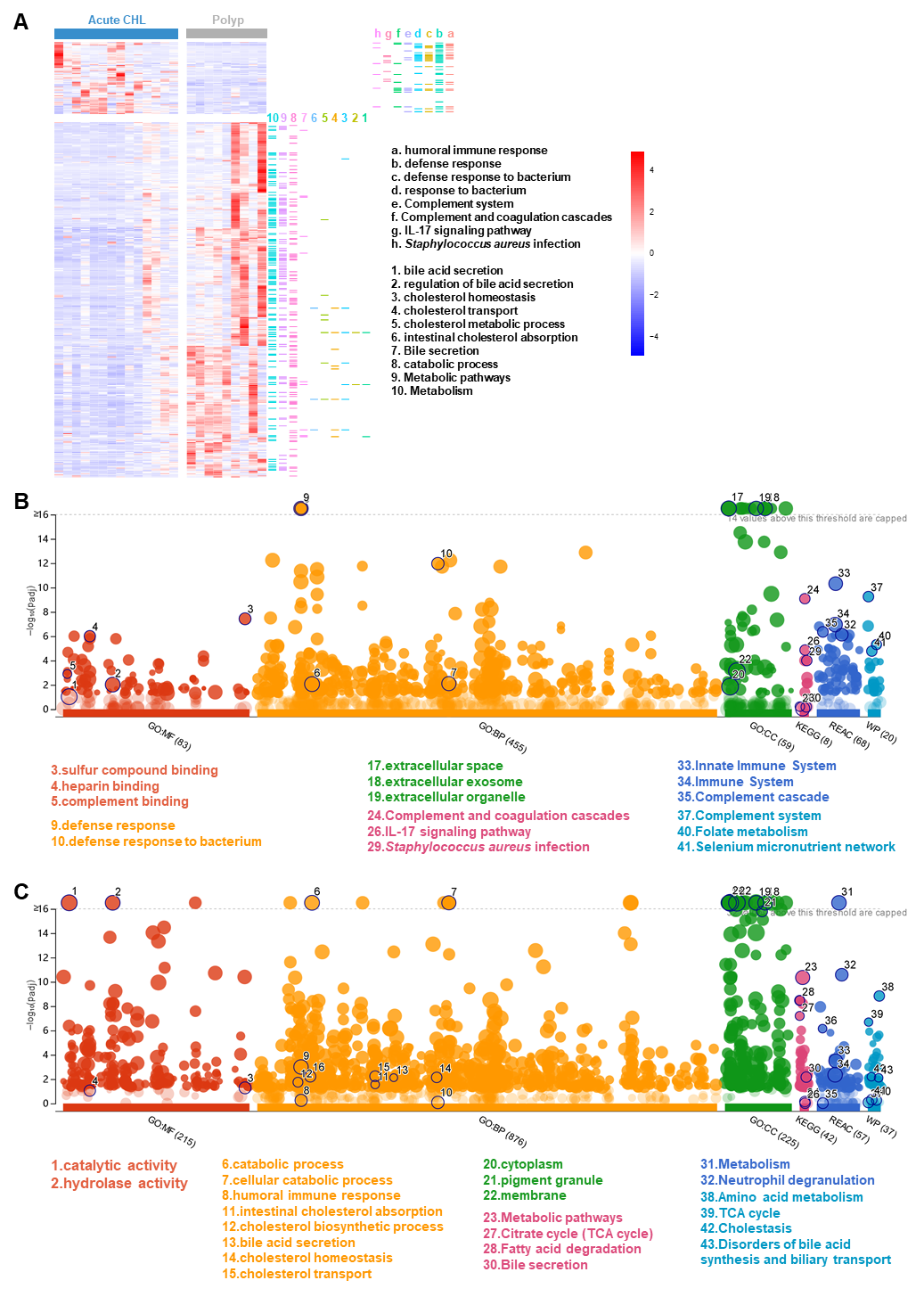


**Supplementary Figure 3.** **Differential human proteins between acute CHL and polyp bile samples. (A**) Hierarchical clustering analysis of differential human proteins between acute CHL (n=14) and polyp (n=9). (**B**) Function enrichment of 74 upregulated human proteins in acute CHL compared to polyp. Function enrichment analysis of differential human proteins was performed using g:Profiler (https://biit.cs.ut.ee/gprofiler/gost) (Raudvere et al., 2019). GO_MF, Gene ontology of molecular function; GO_CC, cellular component; GO_BP biological process; REAC, Reactome; WP, WikiPathways. (**C**) Function enrichment of 362 downregulated human proteins in acute CHL compared to polyp.


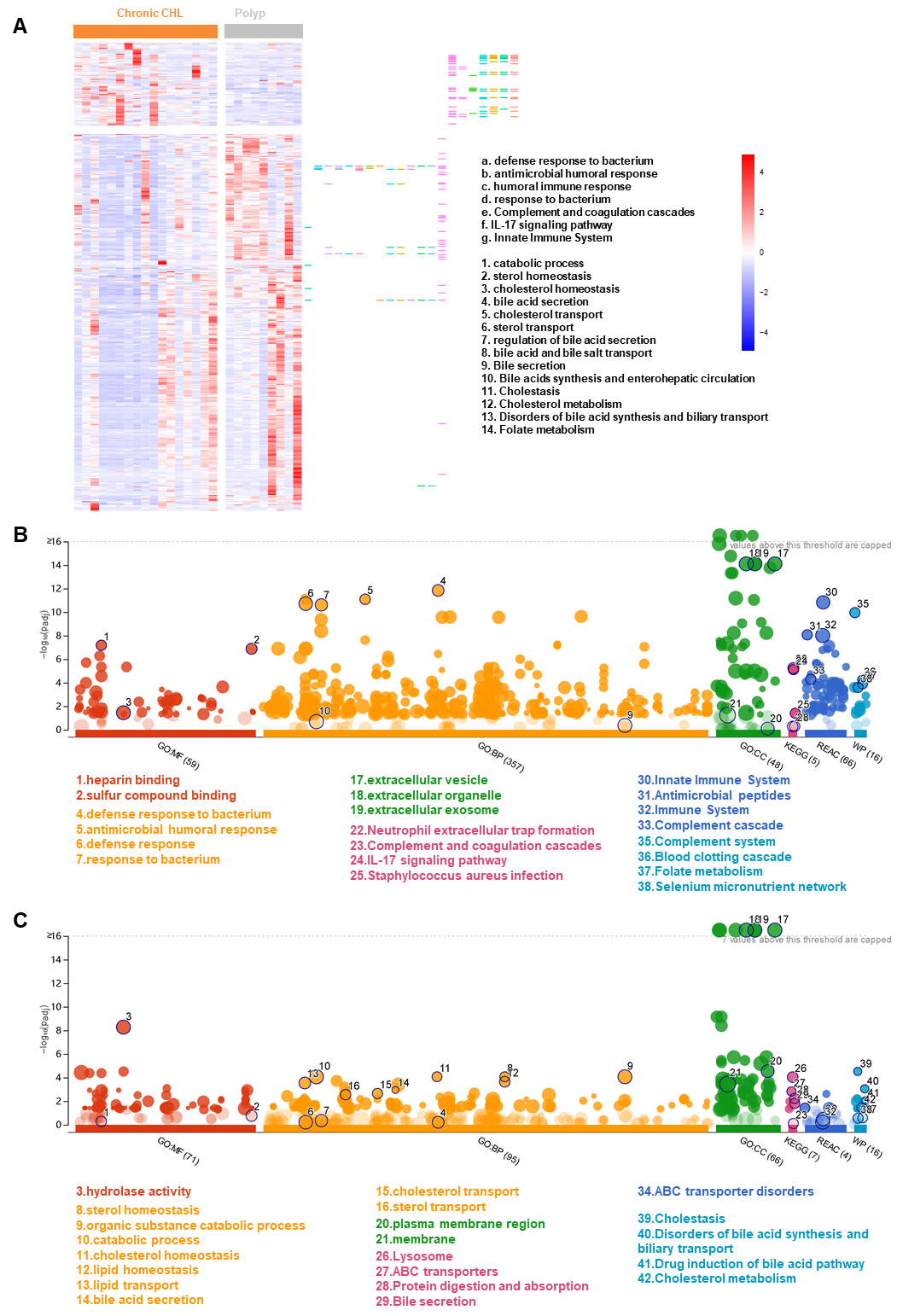


**Supplementary Figure 4.** **Function enrichment of differentially expressed human proteins between chronic CHL and polyp. (A**) Hierarchical clustering analysis of differential human proteins between chronic CHL (n=17) and polyp (n=9). (**B**) Function enrichment of 51 upregulated human proteins in chronic CHL compared to polyp. Function enrichment analysis of differential human proteins was performed using g:Profiler (https://biit.cs.ut.ee/gprofiler/gost) (Raudvere et al., 2019). GO_MF, Gene ontology of molecular function; GO_CC, cellular component; GO_BP biological process; REAC, Reactome; WP, WikiPathways. (**C**) Function enrichment of 95 downregulated human proteins in chronic CHL compared to polyp.


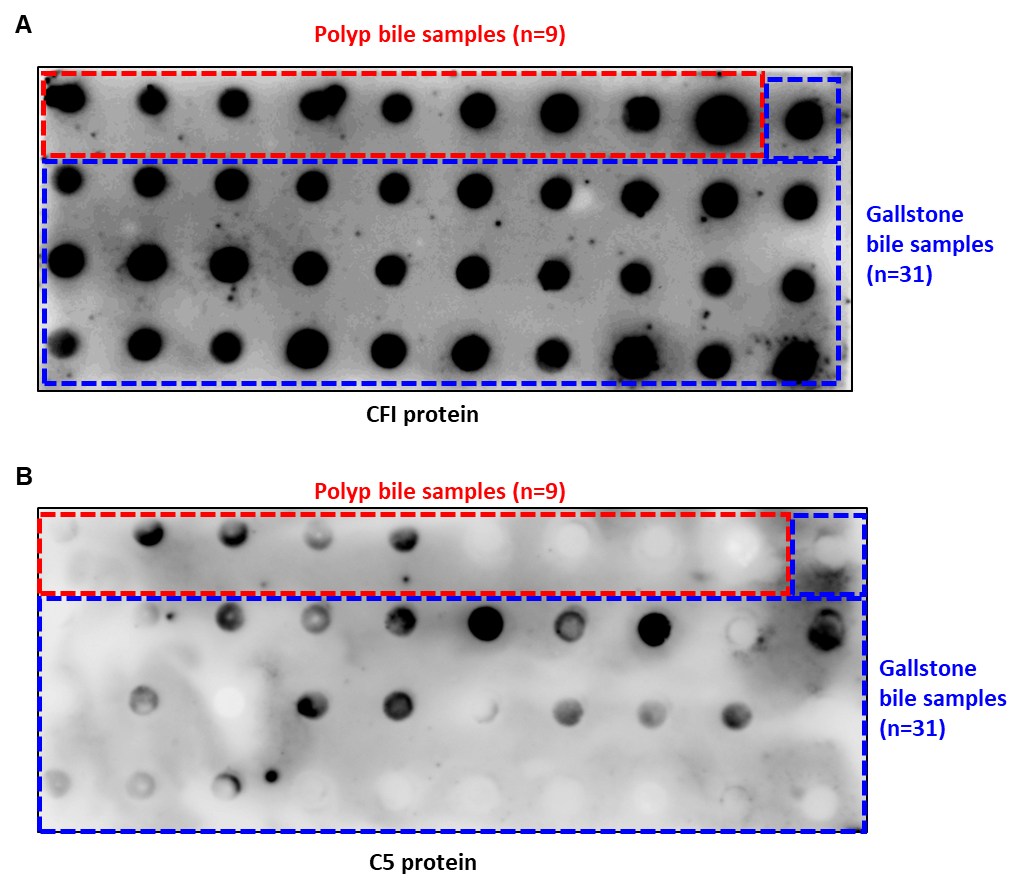


**Figure S5. Validation of complement protein expression by dot blot.** (**A**) Expression of CFI protein was measured in polyp bile samples (n=9, illustrated with a red box) and gallstone bile samples (n=31, illustrated with a blue box) using dot blot. (**B**). Expression of C5 protein was measured in polyp bile samples (n=9, illustrated with a red box) and gallstone bile samples (n=31, illustrated with a blue box) using dot blot.


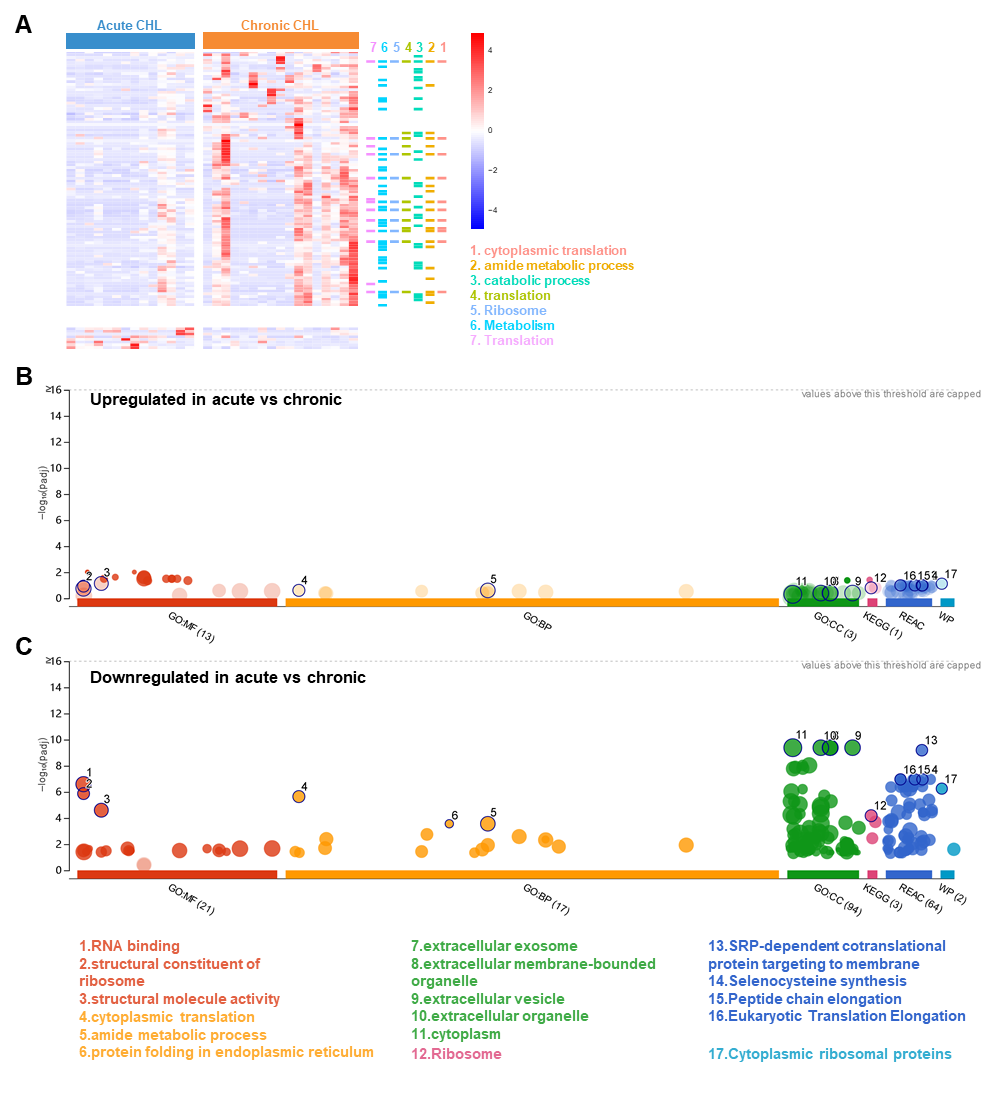


**Supplementary Figure 6.** **Function enrichment of differentially expressed human proteins between acute CHL and chronic CHL. (A**) Hierarchical clustering analysis of differential human proteins between acute CHL (n=14) and chronic CHL (n=17). (**B**) Function enrichment of 8 upregulated human proteins in acute CHL compared to chronic CHL. Function enrichment analysis of differential human proteins was performed using g:Profiler (https://biit.cs.ut.ee/gprofiler/gost) (Raudvere et al., 2019). GO_MF, Gene ontology of molecular function; GO_CC, cellular component; GO_BP biological process; REAC, Reactome; WP, WikiPathways. (**C**) Function enrichment of 95 downregulated human proteins in acute CHL compared to chronic CHL.

**References**

Raudvere, U., Kolberg, L., Kuzmin, I., Arak, T., Adler, P., Peterson, H., et al. (2019). g:Profiler: a web server for functional enrichment analysis and conversions of gene lists (2019 update). *Nucleic Acids Res* 47(W1)**,** W191-W198. doi: 10.1093/nar/gkz369.
